# Transcriptome Analysis on the Inflammatory Cell Infiltration of Nonalcoholic Steatohepatitis in Bama Minipigs Induced by a Long-Term High-Fat, High-Sucrose Diet

**DOI:** 10.1371/journal.pone.0113724

**Published:** 2014-11-21

**Authors:** Jihan Xia, Jing Yuan, Leilei Xin, Yuanyuan Zhang, Siyuan Kong, Yaoxing Chen, Shulin Yang, Kui Li

**Affiliations:** 1 State Key Laboratory of Animal Nutrition, Institute of Animal Sciences, Chinese Academy of Agricultural Sciences, Beijing, P. R. China; 2 College of Animal Science, Yangtz University, Jinzhou, Hubei, P. R. China; 3 College of Veterinary Medicine, China Agricultural University, Beijing, P. R. China; University of Catanzaro Magna Graecia, Italy

## Abstract

Long-term adherence to a high-fat, high-calorie diet influences human health and causes obesity, metabolic syndrome and nonalcoholic steatohepatitis (NASH). Inflammation plays a key role in the development of NASH; however, the mechanism of inflammation induced by over-nutrition remains largely unknown. In this study, we fed Bama minipigs a high-fat, high-sucrose diet (HFHSD) for 23 months. The pigs exhibited characteristics of metabolic syndrome and developed steatohepatitis with greatly increased numbers of inflammatory cells, such as lymphocytes (2.27-fold, P<0.05), Kupffer cells (2.59-fold, P<0.05), eosinophils (1.42-fold, P<0.05) and neutrophils (2.77-fold, P<0.05). High-throughput RNA sequencing (RNA-seq) was performed to explore the systemic transcriptome of the pig liver during inflammation. Approximately 18.2 gigabases of raw sequence data were generated, and over 303 million high-quality reads were assembled into 21,126 unigenes. RNA-seq data analysis showed that 822 genes were differentially expressed in liver (P<0.05) between the HFHSD and control groups. Kyoto Encyclopedia of Genes and Genomes (KEGG) analysis showed that the process of inflammation involved the inflammatory signal transduction-related toll-like receptor, MAPK, and PPAR signaling pathways; the cytokine-related chemokine signaling, cytokine-cytokine receptor interaction, and IL2, IL4, IL6, and IL12 signaling pathways; the leukocyte receptor signaling-related T cell, B cell, and natural killer cell signaling pathways; inflammatory cell migration and invasion- related pathways; and other pathways. Moreover, we identified several differentially expressed inflammation-related genes between the two groups, including FOS, JUN, TLR7, MYC, PIK3CD, VAV3, IL2RB and IL4R, that could be potential targets for further investigation. Our study suggested that long-term HFHSD induced obesity and liver inflammation, providing basic insight into the molecular mechanism of this condition and laying the groundwork for further studies on obesity and steatohepatitis.

## Introduction

Obesity, metabolic syndrome and the associated chronic inflammation are among the most prevalent diseases that impose enormous health and economic burdens on governments around the world and on the global economy [1]. Metabolic disorders of glucose and lipid metabolism in the liver develop into nonalcoholic fatty liver disease (NAFLD) and, often, obesity [2]. Nonalcoholic steatohepatitis (NASH), a key stage of NAFLD, is characterized by hepatic steatosis, inflammation and fibrosis and is emerging as one of the most common liver diseases and a leading cause of cirrhosis [3]. Inflammation is believed to be the driving force behind the development of NASH and also acts as a critical predictor of the histological progression to fibrosis and cirrhosis; thus, inflammation represents a potentially major therapeutic target in NASH [4].

The potential mechanisms of inflammation in NASH include systemic lipotoxicity as a result of over-nutrition, oxidative stress and the production of proinflammatory cytokines, which activate the immune response and drive inflammation [5], [6]. In particular, proinflammatory cytokines such as IL-6 and TNFα act to trigger the diverse hepatic lesions of NASH by inducing hepatic inflammation and fibrosis, which eventually lead to end-stage liver diseases [7]. The interactions of cytokines/growth factors such as IL-2, IL-6, TGF-β, and INF-β to with their receptors initiate various signaling pathways, leading to the activation of multiple transcription factors [8]. Despite the performance of a great deal of previous research, the mechanisms that drive hepatic inflammation during its progression to NASH remain largely unknown. The choice of animal disease model plays an important role in the study of the hepatic inflammation molecular mechanisms of NASH.

Several species have been used as models for obesity and NASH, and rodents have been established as the primary model for these diseases. However, there are metabolic and physiological differences between humans and rodents that have undoubtedly slowed progress and complicated this research [3]. Pigs are rapidly emerging as a biomedical model for obesity and energy metabolism in humans because of their similar metabolic features, cardiovascular systems, and proportional organ sizes [9]. Well-known minipigs, including Ossabaw, Sinclair, Yucatan, Gottingen, and Bama pigs, have been used in the study of obesity as metabolic syndrome models [10]. Therefore, the gene expression changes of NASH in minipigs should provide meaningful reference data for studies in humans. However, no study has analyzed the porcine liver transcriptome in NASH.

Transcriptome analysis provides a large amount of information that can be used to help predict the roles of individual genes and to elucidate the more complex signaling pathways activated in response to external stimuli [11]. RNA-Seq technology has provided a new horizon for the understanding of global gene expression and has demonstrated the complexity of the mammalian transcriptome [12], [13]. The Illumina Genome Analyzer has been widely used as a next-generation sequencing technology and provides powerful alternative strategies for transcriptome analysis using direct RNA-Seq [14]. RNA-Seq technology involves mRNA that has been purified, fragmented, and converted into a cDNA library using adapter sequences [15]. Reads are mapped to a hypothetical gene, allowing a researcher to achieve unprecedented levels of accuracy and specificity when quantifying differentially expressed genes [16]. Previously, only a few reports have used transcriptome analysis in NASH rodents, but none have pursued a RNA-seq analysis of NASH swine livers. Bama minipigs exhibit metabolic syndrome after being fed a high-fat, high-sucrose diet (HFHSD) for 23 months; furthermore, the early signs of NASH, steatosis, oxidation stress, iron overload, lipid peroxidation and cellular damage all can be observed in the liver. In this study, we focused on the genes and mechanisms involved in the response of swine livers to inflammation induced by HFHSD using RNA sequencing analysis (RNA-Seq). The results of this study should be of great importance for the future study and treatment of NASH.

## Materials and Methods

### Animal models and diets

Twelve Bama minipigs of either sex that were six months of age were obtained from the Germplasm Resource Center of Chinese Experimental Minipig, Institute of Animal Sciences, Chinese Academy of Agricultural Sciences. The animals used in this study received humane care according to the criteria outlined in the “Guide for the Care and Use of Laboratory Animals, ISA, CAAS”, and all procedures were approved by the Animal Care and Use Committee of the Germplasm Resource Center of Chinese Experimental Minipig. Six pigs were fed a control diet as the control group, and six were fed a high-fat, high-sucrose diet (53% control diet, 10% pork lard and 37% sucrose) as the HFHSD group. All animals were individually housed in pens under controlled conditions (temperature at 18–22°C, relative air humidity at 30–70%) and fed twice daily on a restricted schedule. They had free access to water for 23 months. At the end of the experiment, the animals were fasted overnight and euthanized with an overdose of ketamine and xylazine. Tissues were immediately frozen in liquid nitrogen and stored at −80°C for subsequent analysis.

### Serology Assay

Body weights were recorded, and blood was obtained after overnight fasting for centrifugation at 3500 rpm for 10 min at 4°C. The serum glucose concentration was assayed using the immobilized glucose oxidase method. The serum insulin concentration was detected in a two-site immunometric assay with monoclonal antibodies as catching and detecting antibodies. Serum triglyceride (TG), total cholesterol (TC), and low density lipoprotein cholesterol (LDL-C) were assessed using an autoanalyzer H7600-020 (Hitachi, Japan).

### NASH Phenotypes

The steatohepatitis inflammation score was calculated as indicated in the NASH Clinical Research Network Scoring System [17]. Liver tissues were fixed in 4% paraformaldehyde and dehydrated in a graded ethanol series for embedding in paraffin. Paraffin tissue sections of 5 µm thickness were stained with hematoxylin and eosin (H&E). On the basis of this H&E staining, the number of inflammatory cells in the livers per unit area was counted. Inflammation was classified as lobular or portal; lobular inflammation was significantly associated with the diagnosis of NASH and scored as 0 (no foci), 1 (<2 foci per 200× field), 2 (2–4 foci per 200× field), or 3 (>4 foci per 200× field). The ultrastructure of the liver was assessed by transmission electron microscopy. Liver samples stored in formaldehyde saline (4%) were cut into 1 mm^3^ pieces and fixed in glutaraldehyde (2.5%) and then postfixed in osmium tetraoxide (2%) and embedded in Spurr's resin. Ultrathin sections were stained with uranylacetate and lead citrate before being visualized under an H-7500 (Japan) transmission electron microscope.

### Sample preparation, read alignment and RNA-seq analysis

The library construction and sequencing were performed at the Shanghai Biotechnology Corporation. The total liver RNA from the six pigs of the HFHSD group and three pigs of the control group was extracted using the Rneasy mini kit (Qiagen, Valencia, USA), and mRNA was isolated with the mRNA Mini Kit. The cDNA libraries were constructed following the RNA Sample Preparation Guide (Illumina, San Diego, CA, USA). The mRNAs were fragmented by incubation in Elute, Prime, Fragment Mix at 94°C for 8 min to obtain 120–200 bp inserts. The first cDNA strand was synthesized with SuperScript II Reverse Transcriptase (Invitrogen, Carlsbad, CA, USA) using random primers, and Ampure XP beads were used to isolate the double-stranded (ds) cDNA synthesized with Second Strand Master Mix. The adapter was ligated to the A-Tailing fragment, and 12 cycles of PCR was performed to enrich those DNA fragments with adapter molecules on both ends, amplifying the amount of DNA in the library. Purified libraries were quantified using a Fluorometer and validated using an Agilent 2100 bioanalyzer to confirm the insert size and to calculate the molar concentration. Clusters were generated with the library diluted to 10 pM and then sequenced on an Illumina Genome Analyzer IIx (GA IIx, Illumina) for 75 cycles for three pigs (144, 146, 159) and 100 cycles for six pigs (120, 126, 138, 140, 157, 161). The reads were collected using the Illumina GA II and sequencing-by-synthesis technology. The sequencing data sets are available at NCBI Sequence Read Archive (SRA) under accession number SRX197296.

### Bioinformatic analysis of the sequence data

During statistical analysis, raw sequence preprocessing and assembly were carried out using the CLC Genomics Workbench program (version 4.9). Preprocessing included the removal of the adapter, low quality terminal bases and ambiguous inner regions. For the de novo sequence assembly, a counting scaffolding algorithm was adopted. The RPKM method was used to calculate the unigene expression in this study. The mapped read counts for each gene were normalized for RNA length and for the total read number in the lane, according to the reads per kilobase of exon per million fragments mapped (RPKM) [18], facilitating the comparison of transcript levels among samples. The clean reads of each library were mapped to the sequences of each unigene. The significance of the differing gene expressions between the experimental group and the control group was determined using the DEGseq and R packages [19]. The false discovery rate (FDR) was applied to identify the threshold of the P value in multiple tests [20]. When the FDR was less than 0.05 and the log2 ratio was greater than 1 (two-fold change), the unigenes were considered to be differentially expressed. Gene abbreviations for the 822 genes exceeding the FDR threshold of P<0.05 were uploaded into the online SAS analysis system to identify potential pathways or networks associated with the dietary treatments. Gene ontology (GO) enrichment analysis and Kyoto Encyclopedia of Genes and Genomes (KEGG) mapping were performed based on the annotation results using an online SAS system of the Shanghai Biotechnology Corporation (enrichment P<0.05). Based on this GO analysis, the unigenes were statistically analyzed and categorized in terms of their cellular components, molecular functions and biological processes.

### Real-time RT-PCR

QRT–PCR was performed to validate the RNA-Seq results for 16 gene transcripts, three pigs (157, 161, 159) of control gruop and six pigs (120, 126, 138, 140, 144, 146) of HFHSD gruop. Total RNA was extracted from the pig livers using Trizol reagent (Invitrogen, Beijing, China) and treated with RNase-free DNase. The PCR primers used in this study are listed in Table 1. First-strand cDNA synthesis was performed using MMLV reverse transcriptase (Promega, Madison, USA) according to the manufacturer's instructions. The PCR reaction was performed on a 7500 real-time PCR System (Applied Biosystems). The reaction conditions were denaturation for 2 min at 95°C, 30 s at 60°C, and 30 s at 72°C. The relative gene expression levels were calculated from the cycle number (Ct value). The relative expression of the gene of interest was analyzed using the 2-ΔΔCT method. Samples were analyzed in triplicate to ensure their statistical significance. Data are shown as average values ± standard deviation, and samples were analyzed in triplicate to ensure statistical significance.

**Table 1:** Primer pairs selected for RNA-seq validation by qRT PCR.

Gene	Forward primer (5′-3′)	Reverse primer (5′-3′)
FADS1	TCAAGTACATGCCATACAACCACCAGC	CATCCAGGCCAAGTCCACCC
CXCL2	CAAACGGAAGTCCTAGCCACTCT	TCAGTTGACTCTGCCATTGTTTAGC
ACSL1	TCTCATGGACTCCTACGGCATCG	CTTCGGTTTCCTTCTGTTGGCTC
APOE5	GCGTGGCTGTAGTTCTGACCTGG	CCGCTGCTCTGGCTGAAGTAGTC
SLA-3	GGGTACAGTCAGGACGCCTACGA	TTCTCATTTGCTCCGCCTCATCG
SLA-1	GATGAGGAGACGCGGAAAGTCAA	TGGTCCCAAGTAGCAGCCAAACA
SLA-8	CATCGTAGGAATCGTTGCTGGTCT	CATCAGAGTTCTCGACGCTGTTGTT
ALDH1B1	AGTTGCCTTCACTGGCTCTACCG	GCCAACACGATGCTCGGACTCTT
INHBC	CCTCCTTCCATTCTGCTGTTCTCA	GTCTTCACCACATTGCCATCACG
MYS	GGAACTTACAACACCCGAGCGACAA	ATCCGAAGGAAATCCAGCGTCCA
ACOX1	ACGGTGAAGAAGATAAGGGAGTTTGGC	CCCTGGCTCAGCAAGGTAGGAATAAA
ECH1	CGCAAGATGATGGCTGACGA	GCGGGAGTAGAGCAGGTTGATT
DHRS4	CCGCCTCCTCGTTTCTCCATCT	TGCAGCCGTCAACCCATTCTT
SORD	CCTCAGGAAATCGCCAATCAAGT	CACAAGTACCAGGGTCCCACCAG
CYP2C18	CCTCGGGACTTCATTGATTGTTT	GAGCCCATATCTCAGGGTGGTAC
CROT	CTTCACCCTGATGCGTTTATTCA	TGGCACCAACTAACTGCTTCTACTG
GAPDH	AGGGCATCCTGGGCTACACT	TCCACCACCCTGTTGCTGTA

### Statistical Analysis

Differential gene expression and bioinformatics analyses were performed using the online SAS analysis system provided by the Shanghai Biotechnology Corporation. All other data were statistically analyzed using the SPSS 13.0 software. Data are shown as average values ± standard deviation, and P<0.05 was considered statistically significant.

## Results

### Obesity, hyperinsulinemia, dyslipidemia, portal and lobular inflammation in the Bama minipig

The HFHSD pigs showed obesity, hyperinsulinemia, dyslipidemia, and portal and lobular inflammation induced by the HFHS diet at 23 months (Table 2). Compared with the control group, the animals in the HFHSD group displayed a significant weight gain (P<0.01). Although the level of blood glucose in the HFHSD pigs did not show a significant difference from controls, their insulin had remarkably increased by 4.96-fold (28.32 vs. 5.71, P<0.001), signifying hyperinsulinemia. The pigs in the HFHSD group exhibited 3.91-fold (1.72 vs. 0.44 mmol/l, P<0.01) higher fasting levels of plasma triglycerides and higher low density lipoprotein cholesterol (LDL-C). There was a significant increase in the number of inflammatory cells, including lymphocytes (13.95±0.017 vs. 6.14±0.012, P<0.05), eosinophils (2.8±0.012 vs. 1.97±0.008, P<0.05), neutrophils (3.80±0.001 vs. 1.37±0.002, P<0.05), and Kupffer cells (5.10±0.015 vs. 1097±0.008, P<0.05), in hepatic lobules in the HFHSD pigs compared with the control group. Inflammation was classified as lobular or portal; all cases of lobular inflammation were significantly associated with a diagnosis of NASH and scored as 0 (no foci), 1 (<2 foci per 200× field), 2 (2–4 foci per 200× field), or 3 (>4 foci per 200× field) [17]. The pigs in the HFHSD group showed whole portal inflammation and mild lobular inflammation in their livers (Table 2).

**Table 2:** Pathological characteristics of the HFHSD and control groups after sacrificing the minipigs.

	HFHSD	Control
Body weight, kg	140.28±8.52**	51.30±5.85
Insulin, uIU/ml	28.32±7.84**	5.71±0.39
Glucose, mmol/l	4.99±1.11	6.62±0.85
Total cholesterol, mmol/l	3.41±0.29**	1.18±0.38
Plasma triglycerides, mmol/l	1.72±0.32**	0.44±0.19
LDL cholesterol, mmol/l	1.32±0.17*	0.62±0.17
**Proportion of inflammatory cells per unit area of the liver**		
Lymphocytes	13.95±0.017*	6.14±0.012
Eosinophils	2.80±0.012*	1.97±0.008
Neutrophils	3.80±0.001*	1.37±0.002
Kupffer Cells	5.10±0.015*	1.97±0.008
**Lobular inflammation**		
0(No foci)	All minipigs	—
1(<2 foci)	—	4 of 6 minipigs
2(2–4 foci)	—	2 of 6 minipigs
3(>4 foci)		
**portal inflammation**	—	All minipigs

Values are means ± standard deviation. * P<0.05 and ** P<0.01 when compared with the control group.

### Liver fatty deposits caused steatosis and inflammatory cell infiltration in the minipigs in the HFHSD group

**Figure 1:**
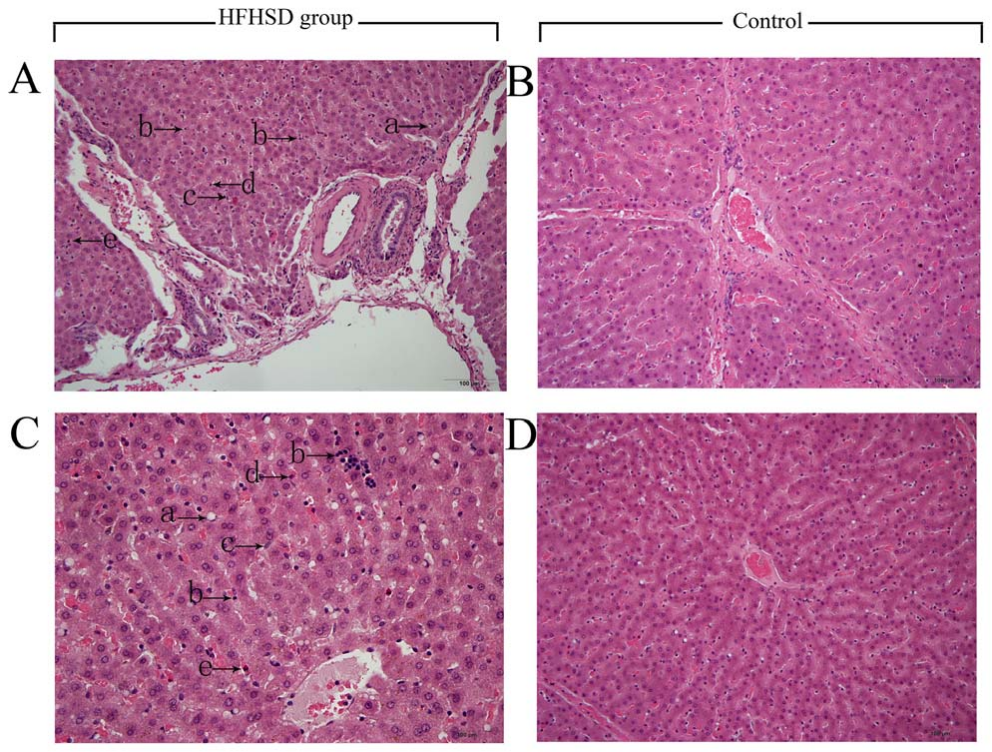
Extensive inflammatory cell infiltration within the dilated hepatic sinusoids in the HFHSD group (H&E stain). A: HFHSD group portal area; B: Control group portal area; C: HFHSD group hepatic lobule; D: HFHSD group hepatic lobule. A, B, C, D, bar  = 100 µm. Note: a, Ito cell; b, lymphocyte; c, Kupffer cell; d, eosinophil; e, neutrophil.

**Figure 2:**
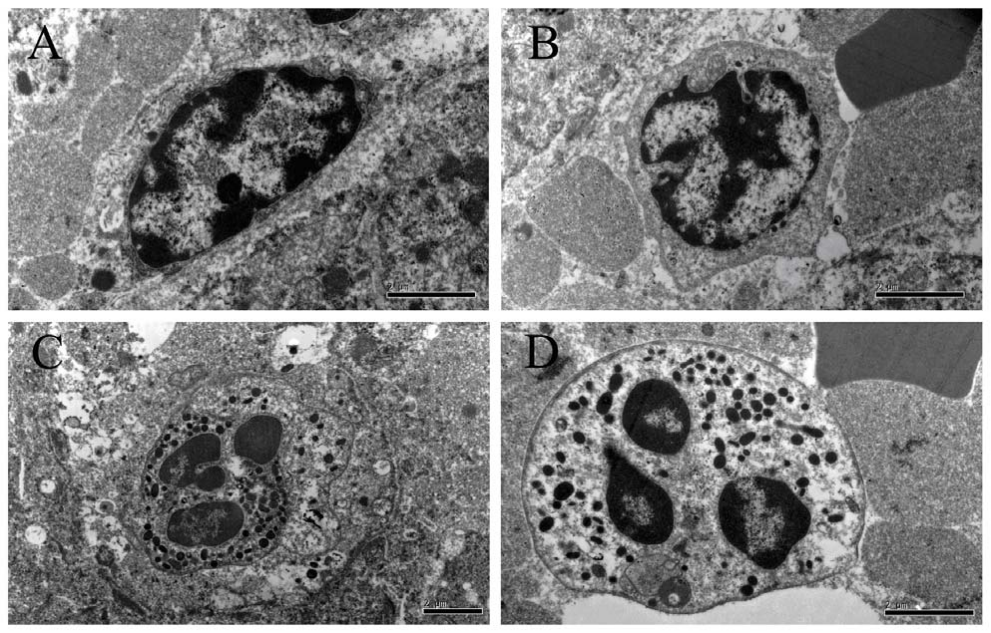
The ultrastructure of inflammatory cells in the hepatic sinusoids of the HFHSD group. There were a large number of phagocytic granules in the Kupffer cells, neutrophils and eosinophils. A: Kupffer cell; B: lymphocyte; C: neutrophil; D: eosinophil; bar  = 2 µm.

The H&E staining results revealed marked steatosis in HFHSD minipig livers compared to the controls (Figure 1). The radial structures of the hepatic cords were very vague and the arrangement of hepatocytes was loose in the HFHSD group compared with the control group. The liver sinusoid was abnormally dilated due to a mixture of severe lobular inflammatory cell infiltration involving lymphocytes, Kupffer cells, eosinophils and neutrophils. Furthermore, the steatosis observed was predominantly microvesicular steatosis (Figure 1). The ultrastructural results indicated that inflammatory cells contained phagocytic granules in the hepatic sinusoids of the HFHSD group (Figure 2). The pigs in the control group did not show hepatic inflammation and appeared normal. Compared with the control group, the HFHSD minipig livers had a significantly higher proportion of inflammatory cells per unit area of liver (Table 2).

### Sequencing and differentially expressed transcripts between the HFHSD and control groups

**Figure 3:**
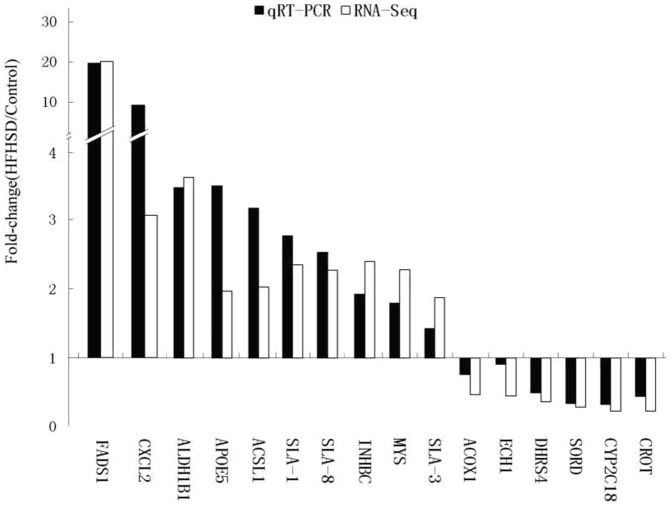
Validation of the RNA-Seq data by qRT-PCR. The 16 genes in minipig livers associated with lipid metabolism, chemokines and the immune response were selected for validation. The data are presented as the fold differences between the HFHSD group and the control group, and each gene was normalized to glyceraldehyde-3-phosphate dehydrogenase (GAPDH) levels.

We sequenced cDNA libraries from 3 (157, 159, and 161) pigs in the control group and 6 (120, 126, 138, 140, 144, and 146) pigs in the HFHSD group using an Illumina GA II at Shanghai Biotechnology Corporation, China. The data set was analyzed according to the Biotechnology bioinformatics protocols for RNA-seq. The sequence reads were submitted to the NCBI Sequence Read Archive under accession number SRX197296. In total, we acquired 302.6 million reads from nine cDNA pig libraries, comprising 18.2 gigabases (Gb) of cDNA sequence (Table 3). Tolerances were set to allow at most two mismatches for 60 bp reads in each alignment. Approximately 82% of the sequenced reads (303 million mapped reads) were successfully aligned to the swine genome reference sequence (SGSC SusScr2). Furthermore, the expression patterns of 16 randomly selected transcripts between two individuals were validated by qRT- PCR. The technical replicates and qRT-PCR data confirmed the repeatability and reproducibility of the gene expression data obtained by RNA sequencing (Figure 3).

**Table 3:** Summary of the numbers of total reads, matched genes and junction reads of each individual in this study.

Sample	120	126	138	140	144	146	159	157	161
**Total reads**	36,360,909	32,028,695	30,502,022	35,724,335	33,368,910	33,157,592	35,378,945	35,220,261	30,907,575
**Total unmapped reads**	4,251,214	5,332,896	6,860,322	7,790,108	6,640,985	7,177,759	8,575,552	4,000,152	5,308,881
**Reads perfectly matched to the reference genome**	25,686,238	20,343,808	18,289,842	21,494,995	20,294,336	20,073,970	20,013,908	24,915,261	19,375,043
**Reads with < = 2 bp mismatch to the reference genome**	32,109,695	26,695,799	23,641,700	27,934,227	26,727,925	25,979,833	26,803,393	31,220,109	25,598,694
**Reads uniquely matched to the reference genome**	25,986,037	21,373,649	19,021,200	22,378,551	22,309,584	21,875,698	22,493,301	25,388,184	20,612,013
**Reads matched to the reference genome with multi-positions**	2,836,393	2,452,487	2,131,677	2,551,157	2,040,594	1,895,355	1,979,763	2,690,412	2,312,202
**Junction reads**	4,096,064	3,577,367	2,847,393	3,264,879	3,720,769	2,751,876	3,004,393	3,849,199	3,065,877

The differentially expressed genes were selected based on their expression profiles and the following criteria: (1) the fold change in gene expression levels between the two groups was greater than or equal to two (log_2_ fold change ≥1 or ≤−1); (2) the false discovery rate value was less than 0.05; and (3) the P-value was less than 0.05. We identified 822 differentially expressed genes out of a total 24,126 genes; of these, the expression of 430 genes was up-regulated, and the expression of 392 genes was down-regulated.

**Figure 4:**
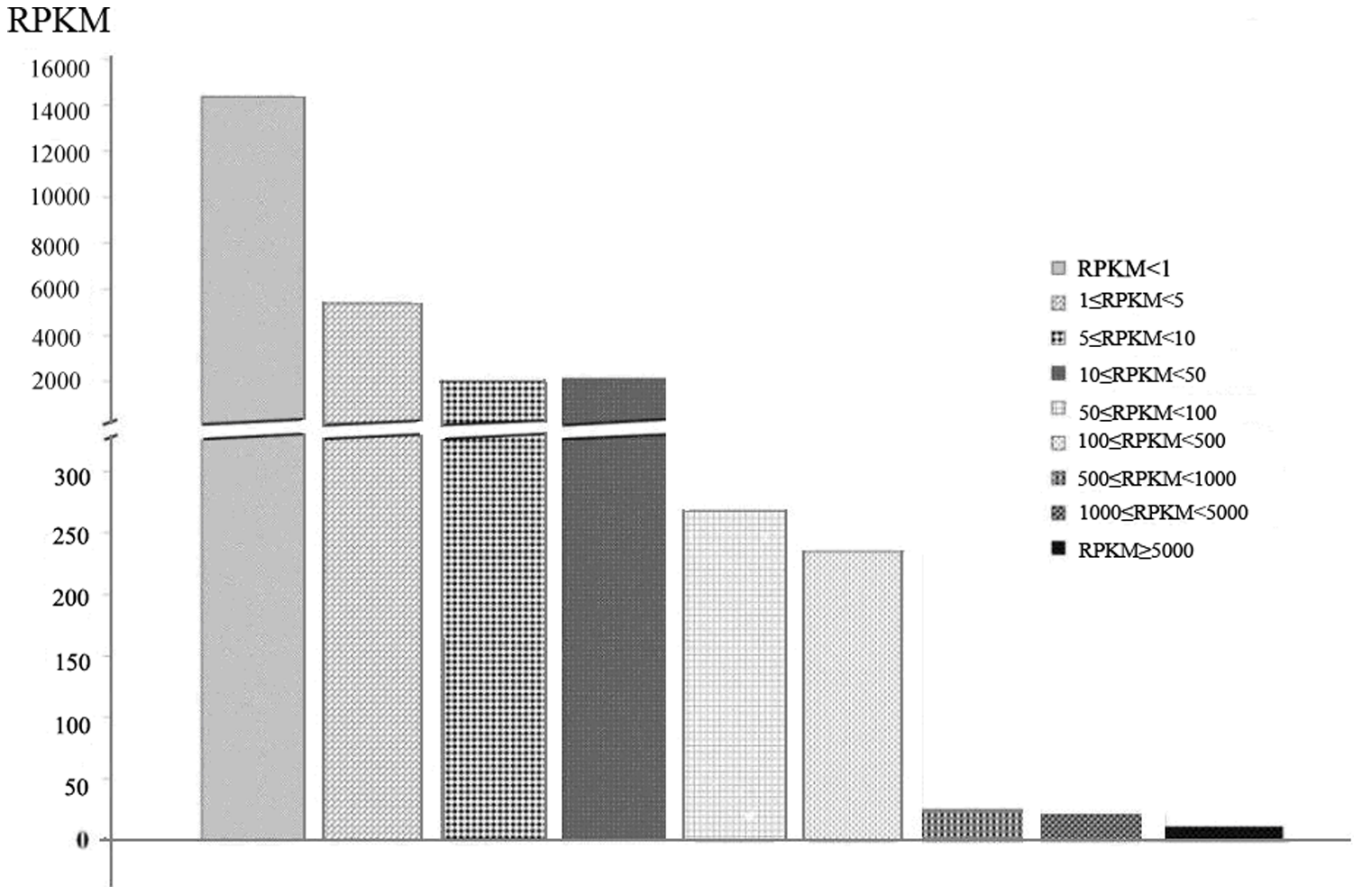
Distribution of the genes according to their expression profiles by RPKM.

In this study, a total of 24,126 genes were found to be expressed in pig livers. The RPKM method was used to calculate the unigene expression in this study. The mapped read counts for each gene were normalized for RNA length and the total read number in the lane, according to RPKM, in a procedure that facilitates the comparison of transcript levels among samples. The numbers of genes for RPKM<1, 1≤RPKM<5, 5≤RPKM<10, and 50≤RPKM<100 were 14,370, 5472, 1801, and 1918, respectively. When RPKM was larger than 50, the number of genes decreased rapidly. The number of genes for 50≤RPKM<100, 100≤RPKM<500, 500≤RPKM<1000, 1000≤RPKM<5000, and RPKM≥5000 were 269, 236, 26, 22, and 12, respectively. Thus, the greatest number of genes for RPKM was noted at less than 50 (Figure 4).

**Figure 5:**
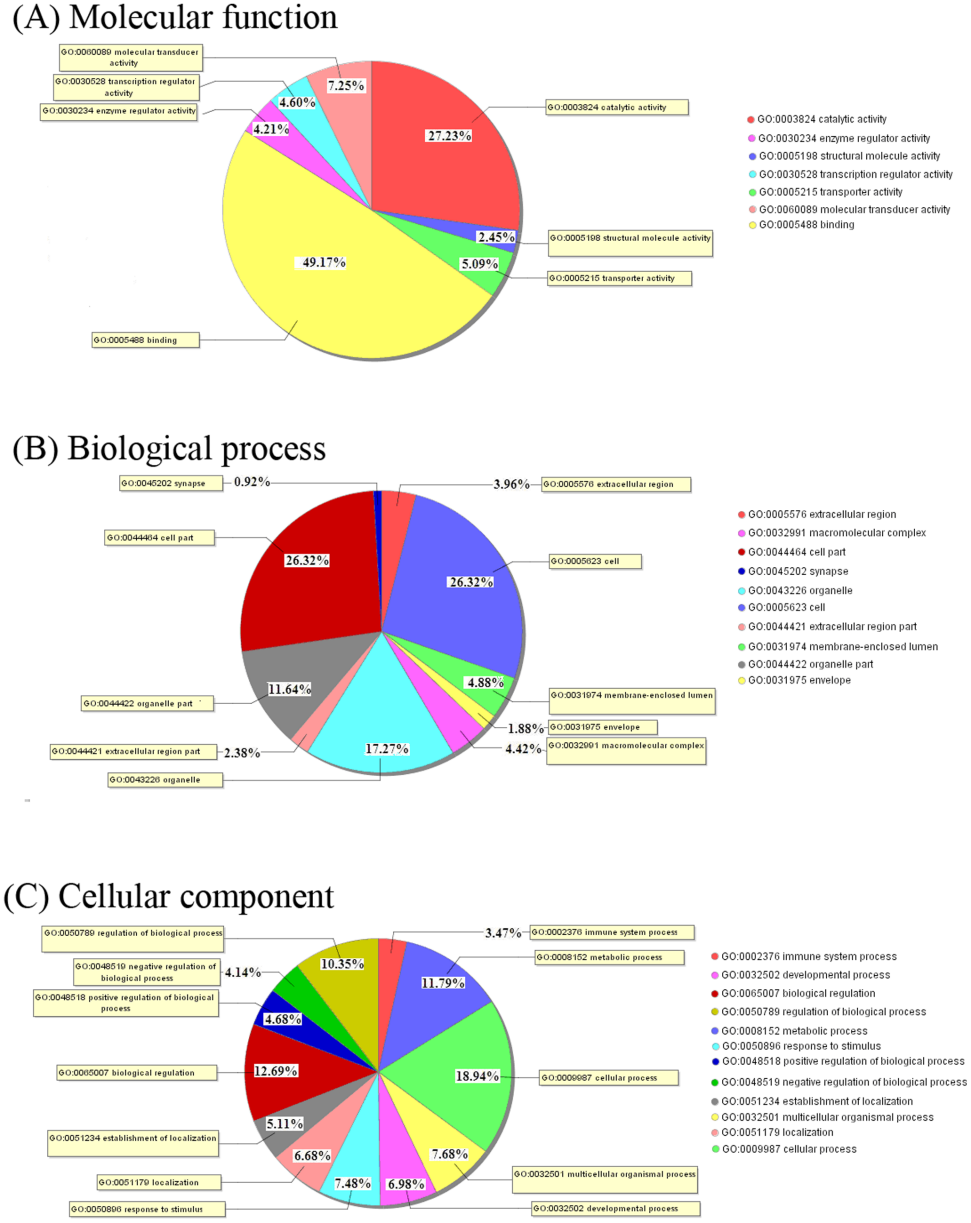
Gene ontology (GO) analysis of the Bama minipig transcriptome data (GO level 2). GO analysis of 822 genes differentially expressed between the HFHSD group and the control group, which was used to predict the genes' involvement in biological processes (A), molecular functions (B) and cellular components (C). The GO Id and name are shown with different colors.

To further investigate the biological relationships of differentially expressed genes and their phenotypes, we performed a GO analysis by entering each differentially expressed gene into the records of the GO database. The results of these GO functional annotations are presented in Figure 5 and Table S1. Because some of the genes were assigned to multiple GO terms, the total number of genes in the three GO terms was greater than that of the differentially expressed genes. The genes were classified as follows: 617 genes mapped to biological process terms, 670 genes mapped to cellular component terms, and 617 genes mapped to molecular function terms. Among the molecular function assignments, a high percentage of genes were assigned to binding (49.17%) and catalytic activity (27.23%). As shown in Figure 5, for the cellular component term, the differently expressed genes were classified under cellular processes (18.94%), metabolic (11.79%), and biological regulation (12.69%), which contained those genes involved in the basic processes for the survival of an organism. We were especially interested in immune system processes and the signaling and regulation of biological processes. However, the present GO assignments and distributions are only the results of theoretical prediction based on homology searches and should be verified through experimental procedures [21].

### Analysis of the inflammation-related genes and pathways in NASH minipig livers

All 822 of the differentially expressed genes were imported into the SBC Analysis System, including the BioCarta and Kegg databases, to identify relevant pathways. Several pathways were identified as potentially having been affected by the HFHSD diet (p<0.05). The pathways related to inflammatory signal transduction and inflammatory cell infiltration are activated significantly. First, hepatocyte injury caused the activation of inflammatory signal transduction-related pathways, including the toll-like receptor pathway (P<0.005), the MAPK signaling pathway (P<0.01), and the PPAR signaling pathway (P<0.01). Second, chemokines and cytokines that were secreted by leukocytes regulate the immune system and related pathways, including the chemokine signaling pathway (P<0.01), the cytokine-cytokine receptor interaction pathway (P<0.01), and the IL2, IL4, IL6, IL7, and IL12 signaling pathways (P<0.001). Furthermore, leukocyte receptor signaling pathway-activated leukocytes contribute to the reaction to the detriment of the liver; these pathways include the T cell receptor signaling pathway (P<0.001), the B cell receptor signaling pathway (P<0.001), and natural killer cell-mediated cytotoxicity (P<0.01). In addition, leukocytes interacted with endothelial cells and passed through the blood vessels into liver lobules by several pathways, such as the regulation of actin cytoskeleton pathway (P<0.01) and leukocyte transendothelial migration (P<0.01). Through the analysis of the RNA-Seq data pathways, we investigated the process of inflammatory cell infiltration and obtained more detailed pathway information (Table S2).

## Discussion

### Long-term over-nutrition-induced inflammation in Bama minipigs

NASH is invariably associated with over-nutrition, obesity, insulin resistance, and metabolic syndrome. Inflammation is considered the driving force behind the development of nonalcoholic steatohepatitis, the progression to fibrosis and subsequent cirrhosis [22]. In our study, a long-term high-fat, high-sucrose diet induced NASH in Bama minipigs, simulating the disease process of human over-nutrition. After 23 months, the groups of HFHSD pigs exhibited enormous obesity, hyperinsulinemia, dyslipidemia, marked steatosis, obvious portal inflammation and mild lobular inflammation, accompanied by significantly increased inflammatory cells and significantly increased lymphocytes (2.27-fold, P<0.05), Kupffer cells (2.59-fold, P<0.05), eosinophils (1.42-fold, P<0.05) and neutrophils (2.77-fold, P<0.05). In a human NAFLD study, inflammation in NAFLD was found to usually consist of a mixed inflammatory cell infiltrate composed of lymphocytes, plasmacytes, monocytes, macrophages and neutrophils [23]. Most of the inflammatory cell infiltrate in NASH is composed of lymphocytes, and their number varies depending on the severity of necroinflammatory injury [24]. NASH diagnosis supports the significant role of Kupffer cells in the pathogenesis and progression of NAFLD by regulating triglyceride storage, mediating the inflammatory response, and contributing to hepatocyte injury [25]. Neutrophils are an important source of oxidative stress-related molecules [26], although different types of mononuclear cells are also present. Additionally, natural killer T cells are regulated by T lymphocytes and are considered to be innate immune effectors [27]. Activated Kupffer cells and hepatic stellate cells also contribute significantly to cytokine expression during NASH [28]. In summary, the inflammation found in Bama minipigs in this study was essentially similar to that of human NASH and may contribute to the further study of NASH pathology and molecular mechanisms. Among the interesting findings, the process of liver inflammatory cell inflation was observed through the analysis of RNA-seq data, which contained differentially expressed pathways and genes, including the transduction of inflammatory signals, the secretion of chemokines and cytokines, the activation of leukocyte and inflammatory cell invasion. Detailed information regarding the observed changes in the genes and pathways is given in Table S3.

### Inflammation signal transduction and cytokine secretion in the HFHSD minipig livers

**Figure 6:**
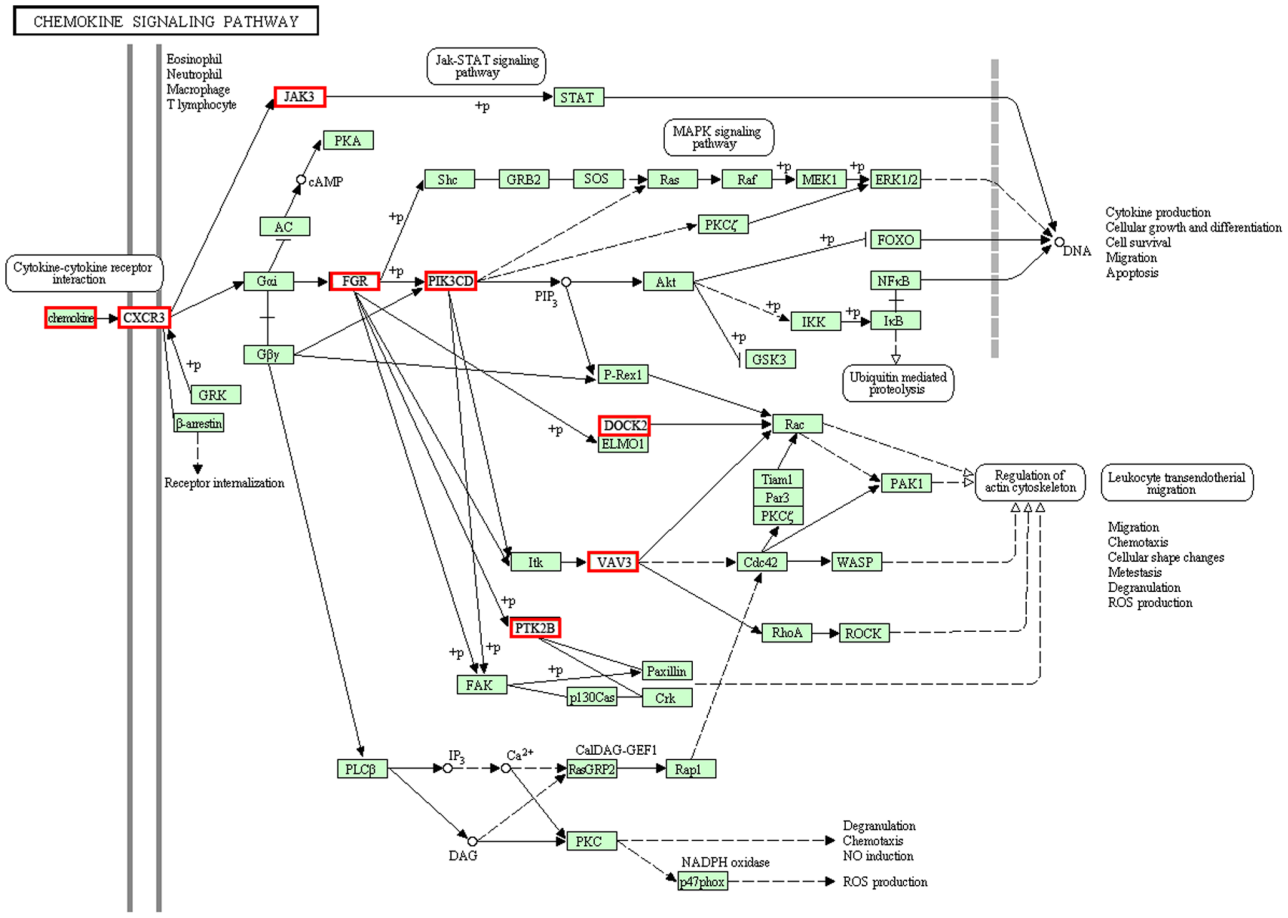
List of genes involved in the chemokine signaling pathway. The genes in the red box represent differentially expressed genes between the HFHSD group and the control group in RNA-seq analysis. All other genes had no obvious differences in this experiment.

Over-nutrition and lipid dysfunction contributed to endoplasmic reticulum stress, oxidation, cellular injury, and, ultimately, inflammation, consisting of signal transduction through the toll-like receptor, MAPK, and PPAR signaling pathways. The toll-like receptor pathway was implicated in various molecular models of disease, recognizing endogenous substances to transmit inflammatory signals. The PPAR and MAPK pathways can also play an important role in the development of NASH. The PPAR pathway regulates various inflammatory responses using agonist-dependent mechanisms to affect the levels of transcription factors such as AP1, STATs, and NFAT [29]. The binding of pro-inflammatory cytokines to their receptors triggers the mitogen-activated protein kinase (MAPK) pathway and ultimately results in the activation of transcription factors, including nuclear factor kappa B and the c-Jun part of activating protein-1. These transcription factors activate the expression of a wide variety of genes, including cytokines, chemokines, and adhesion molecules, to recruit leukocytes to the sites of inflammation [30]. Accompanied by the profoundly increased expression of proinflammatory and immune system factors, such as IL-2, IL-4, IL-6, IL-7, and TGF-β, the immune system is regulated after liver injury. Chemokines are small heparin-binding proteins that direct the movement of circulating leukocytes to the site of inflammation or injury, as shown in Figure 6 [31]. In the group of live HFHSD pigs, the chemokine signaling pathways of leukocytes were activated; these pathways are responsible for the regulation of the cytoskeleton, transendothelial migration, cytokine production, cell growth and differentiation, and leukocyte aggregation to clear the products of tissue damage (Figure 6, Table S3).

These chemokines bind the receptor CXCR3, a G protein-coupled receptor, to induce cellular responses [32]. The signal from CXCR3 is transmitted to FGR and PIK3CD, the former of which is involved in mediating the protein–protein interactions with phosphotyrosine-containing and proline-rich motifs and acts as a regulator of cell migration and adhesion [33]. PIK3CD is involved in signal transduction, primarily in leukocytes [34], and acts on the genes DOCK2, VAV3 and PTK2B. The protein encoded by the DOCK2 gene belongs to the CDM protein family and is involved in the remodeling of the actin cytoskeleton required for lymphocyte migration through the activation of RAC [35]. VAV3, a Rho family GTPase guanine nucleotide exchange factor (GEF), activates the pathways leading to actin cytoskeletal rearrangements and transcriptional alterations [36]. The chemokine signaling pathway is involved in leukocyte trafficking, cytoskeletal changes and chemotactic migration, which were caused by ROS and cytotoxicity [24].

### Activation of the lymphocyte signaling pathway contributed to the enhancement of inflammation in the HFHSD minipig livers

**Figure 7:**
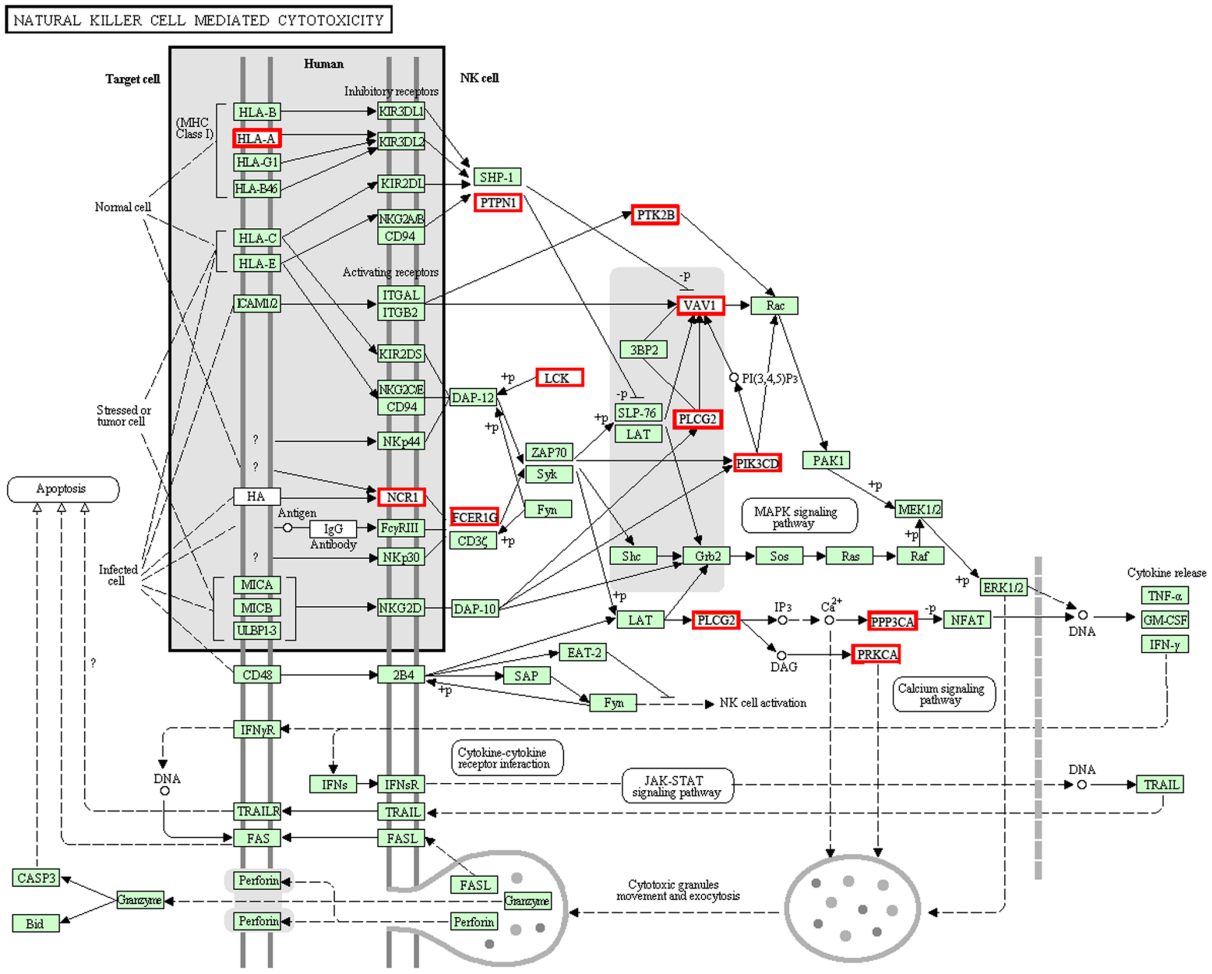
List of genes involved in the natural killer cell-mediated cytotoxicity pathway. The genes in the red box illustrate differentially expressed genes between the HFHSD group and the control group in the RNA-seq analysis. All other genes had no obvious differences in this experiment.

**Figure 8:**
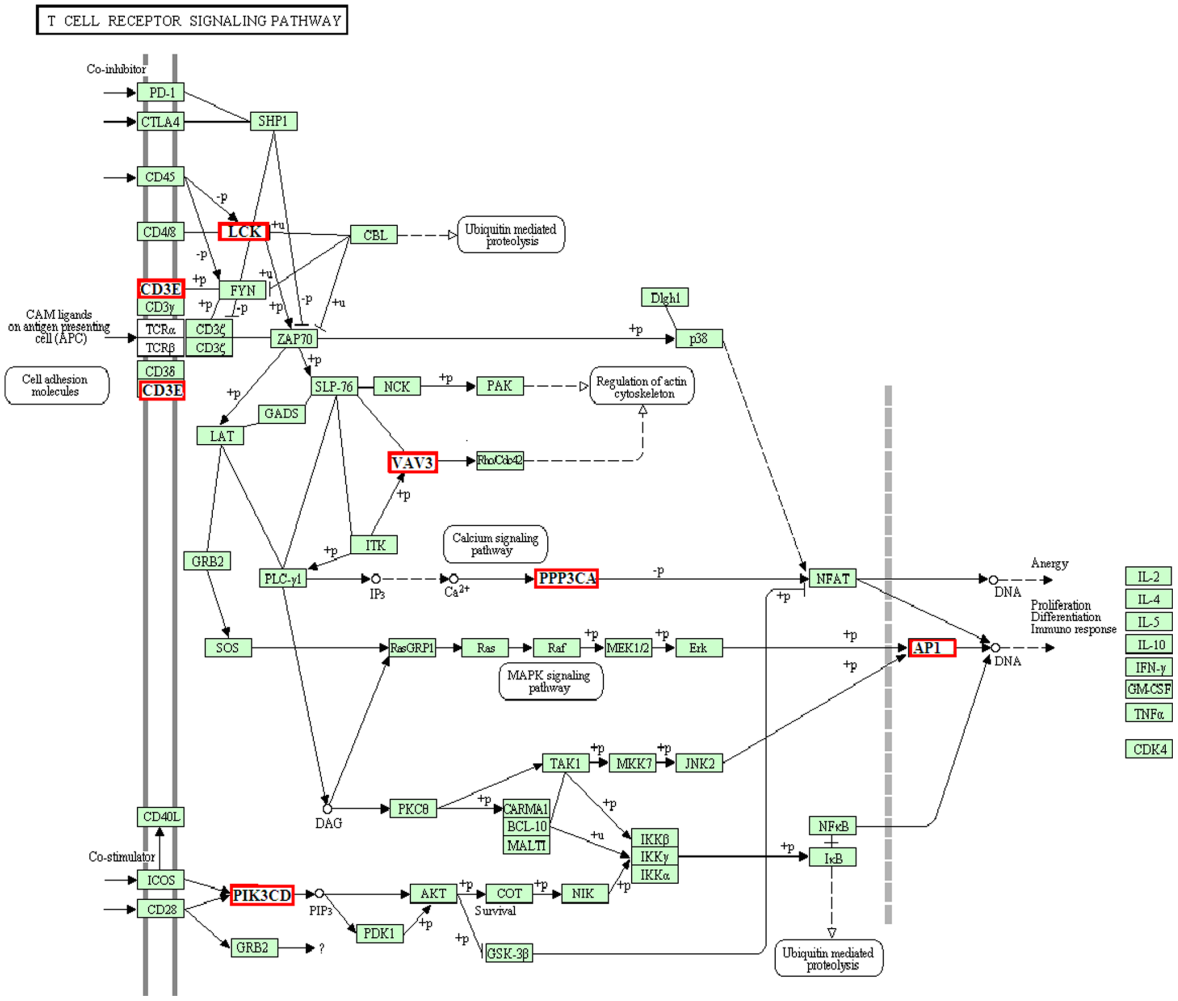
List of genes involved in the T cell receptor signaling pathway. The genes in the red box represent differentially expressed genes between the HFHSD group and the control group in the RNA-seq analysis. All other genes had no obvious differences in this experiment.

Over the 23-month induction process, oxidative stress and lipotoxicity caused inflammation, resulting in chronic damage to the liver tissues. The levels of leukocytes, such as T cells, B cells, NK cells, Kupffer cells, eosinophils and neutrophils, increased significantly as these cells accumulated in the HFHSD minipig livers (Table 2, Figure 1). A great up-regulation of the genes related to the increase in inflammatory cells in the HFHSD group was noted. After analyzing the inflammatory pathways, we can clearly see that inflammatory cells such as T cells, B cells and natural killer cells were activated in the HFHSD minipig livers. Two pathways in particular are important in the progression of inflammation, as shown in Figure 7 and Figure 8.

Natural killer cells are important immune cells that can directly kill target cells involved in immune regulation. In the HFHSD minipig livers, the natural killer cells cleared away damaged hepatocytes and cytotoxic granules; further released cytokines such as TNF, CSF, and IFN; and were involved in the regulation of liver inflammation (Figure 7, Table S3). The cell membrane proteins NCR1 and HLA-A received the extracellular signal and then transmitted it to PTPN1 and FCER1G. HLA-A belongs to the HLA class I heavy chain paralogues, which are anchored in the membrane and play a central role in the immune system by presenting signals [37]. Additionally, PTPN1 is the founding member of the protein tyrosine phosphatase (PTP) family, which catalyzes the hydrolysis of phosphate monoesters, specifically on tyrosine residues [38]. FCER1G, a high affinity IgE receptor, is a key molecule involved in signal transmission. The signal finally passes through the genes PPP3CA and PRKCA and past the middle signal molecules, PTK2B, VAV1, PLCG2 and PIK3CD. PTK2B encodes a cytoplasmic protein tyrosine kinase that is involved in rapid tyrosine phosphorylation and activation in response to increases in intracellular calcium concentration, membrane depolarization, or protein kinase C activation [39]. The VAV1 protein is a guanine nucleotide exchange factor that activates pathways leading to actin cytoskeletal rearrangements and transcriptional alterations. This protein should be important in hematopoiesis due to its effects on T-cell and B-cell development and activation [40]. The protein encoded by PLCG2 is a transmembrane signaling enzyme that catalyzes the conversion of IP3 and diacylglycerol (DAG). IP3 and DAG are second messenger molecules important for transmitting signals from immune system receptors across the cell membrane. Therefore, the natural killer cell-mediated cytotoxicity pathway played an important role in the release of cytokines and in the movement and exocytosis of cytotoxic granules in the pigs of the HFHSD group.

T cells are also important immune cells that directly kill target cells and release cytokines such as IL-2, IL-4, and IL-10. In the HFHSD minipig livers, the activation of the T cell receptor signaling pathway caused cytokine release and inflammation. The signal was received by CD3E and then transmitted to molecular AP1 by the molecular intermediaries LCK, PPP3CA, VAV3 and PIK3CD (Figure 8, Table S3). CD3E is an important molecule in intracellular signal-transduction pathways that codes for a component of the T-cell receptor-CD3 complex [41]. The gene LCK, which encodes a protein present in tyrosine kinases, is a key signaling molecule that is involved in mediating protein–protein interactions with phosphotyrosine-containing and proline-rich motifs [42]. PIK3CD is involved in immune response, and its protein product is a class I PI3K found primarily in leukocytes. In the HFHSD pigs, the T cell signaling pathway activated T cells, an immune reaction, leukocyte proliferation and inflammation regulation.

### Various inflammatory cells migrated transendothelially and invaded the hepatic lobules of Bama minipigs in the HFHSD group

**Figure 9:**
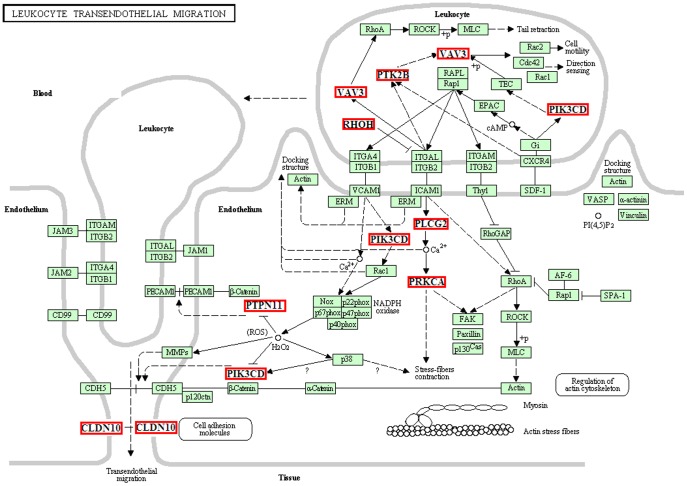
List of genes involved in the leukocyte transendothelial migration pathway. The genes in the red box illustrate differentially expressed genes between the HFHSD group and the control group in the RNA-seq analysis. All other genes had no obvious differences in this experiment.

A large number of leukocytes perforated the vessel walls from the blood to the hepatic lobules, thereby leading to inflammation in the HFHSD pig livers (Figure 1). Due to the cell injuries caused by ROS and lipotoxicity, a large number of leukocytes invaded the liver from the blood. We found that the leukocyte transendothelial migration pathway showed a significant difference between the groups, and many related genes are involved in this process, which spans the two essential stages of cell motility and endothelial contraction. First, PIK3CD and PTK2B activated the gene VAV3, and VAV3 activated the downstream target gene for leukocyte motility and direction sensing (Figure 9, Table S3). Increasing intracellular calcium concentration, membrane depolarization, or protein kinase C activation caused rapid PTK2B-driven tyrosine phosphorylation and activation. The VAV3-activated pathways led to actin cytoskeletal rearrangements, impacting leukocyte motility, and to transcriptional alterations. Second, the endothelium enlarged the intercellular space via the contractions of fibers and cytoskeletal regulation for leukocyte motility. PLCG2 catalyzed the conversion of IP3 and diacylglycerol (DAG) using calcium as a cofactor. IP3 and DAG act as second messenger molecules, transmitting signals across the cell. Furthermore, PLCG2 enhanced the calcium concentration and activated PRKCA, a family of serine- and threonine-specific protein kinases that induce the contraction of stress fibers to facilitate the transendothelial migration of leukocytes by passing through the paracellular space between the epithelial or endothelial cell sheets [43]. The change in CLDN10, which is an integral membrane protein and is a tight junction strand component, facilitated the crossing of leukocytes through the blood vessels. Tight junction strands serve as a physical barrier to prevent solutes and water from passing freely through the paracellular space between epithelial or endothelial cell sheets and play a critical role in maintaining cell polarity and signal transduction [44].

## Conclusions

In summary, the findings of the current study provide the first direct support for the long-term induction of NASH by a high-fat, high-sucrose diet in Bama minipigs. The minipigs exhibited obesity, hyperinsulinemia, dyslipidemia, and inflammation accompanied by significantly increased levels of inflammatory cells. In the present analyses of the Bama minipig liver transcriptome, differences in genes and pathways clearly indicated progressive signal transduction, including the chemokine signaling pathway, the TGF signaling pathway, and the cytokine receptor interaction pathway, directed toward the invasion of inflammatory cells, including T cells, B cells, and natural killer cells, and toward leukocyte transendothelial migration. However, further investigation into the molecular mechanisms and potential inflammatory processes associated with NASH is warranted.

## Supporting Information

Table S1 **Detailed information on the GO enrichment analysis of differentially expressed unigenes.**
(XLS)

Table S2 **Detailed information on the pathway enrichment analysis of differentially expressed unigenes.**
(XLS)

Table S3 **Genes involved in the inflammation-related pathway induced by the high-fat, high-sucrose diet in the liver.**
(XLS)
